# Supplementary material for: Exceptionally Fast Temperature-Responsive, Mechanically Strong and Extensible Monolithic Non-Porous Hydrogels: Poly(N-isopropylacrylamide) Intercalated with Hydroxypropyl Methylcellulose
Source: Gels. 2023 Nov 24;9(12):926. doi: 10.3390/gels9120926 (PMC10742870; doi:10.3390/gels9120926)
Supplement: Supplementary file 1 [file gels-09-00926-s001.zip › gels-2723129-supplementary.pdf]

SUPPLEMENTARY INFORMATION

Exceptionally Fast Temperature-Responsive, Mechanically Strong and Extensible Monolithic Non-Porous Hydrogels: Poly(N-isopropylacrylamide) Intercalated with Hydroxypropyl Methylcellulose

Beata Strachota[1], Adam Strachota[1], Leana Vratović[1], Ewa Pavlova[1], Miroslav Šlouf[1], Samir Kamel[2], Věra Cimrová[1]

[1] Institute of Macromolecular Chemistry, Academy of Sciences of the Czech Republic, Heyrovskeho nam. 2, 162 00 Praha, Czech Republic

[2] Cellulose and Paper Department, National Research Centre, 33, El-Bohouth Str., Dokki, Giza 12622, Egypt

1 Synthesis

Table S1 *Hydrogel synthesis: amounts of the reaction mixture components.*

Sample name	water (g)	HPMC (g)	HPMC content in dry gel (wt.%)	NIPA (g)	NIPA (mmol)	BAA (g)	BAA (mmol)	TEMED (g)	TEMED (mmol)	1% APS solution (g)	APS (mmol)
1B	4.538	0.000	0.0	0.5	4.42	0.0034	0.022	0.014	0.120	0.8861	0.039
1B-1H	4.538	0.060	10.6	0.5	4.42	0.0034	0.022	0.014	0.120	0.8861	0.039
1B-2H	4.538	0.121	19.4	0.5	4.42	0.0034	0.022	0.014	0.120	0.8861	0.039
1B-3H	4.538	0.184	26.8	0.5	4.42	0.0034	0.022	0.014	0.120	0.8861	0.039
1B-5H	4.538	0.313	38.3	0.5	4.42	0.0034	0.022	0.014	0.120	0.8861	0.039
2B	4.5859	0.000	0.0	0.5	4.42	0.007	0.045	0.0147	0.126	0.8951	0.0392
2B-1H	4.5859	0.0605	10.7	0.5	4.42	0.007	0.045	0.0147	0.126	0.8951	0.0392
2B-2H	4.5859	0.122	19.4	0.5	4.42	0.007	0.045	0.0147	0.126	0.8951	0.0392
2B-3H	4.5859	0.186	26.8	0.5	4.42	0.007	0.045	0.0147	0.126	0.8951	0.0392
2B-5H	4.5859	0.316	38.4	0.5	4.42	0.007	0.045	0.0147	0.126	0.8951	0.0392
4B	4.685	0.000	0.0	0.5	4.42	0.0142	0.092	0.015	0.128	0.9138	0.040
4B-1H	4.685	0.062	10.8	0.5	4.42	0.0142	0.092	0.015	0.128	0.9138	0.040
4B-2H	4.685	0.125	19.6	0.5	4.42	0.0142	0.092	0.015	0.128	0.9138	0.040
4B-3H	4.685	0.189	26.9	0.5	4.42	0.0142	0.092	0.015	0.128	0.9138	0.040
4B-5H	4.685	0.322	38.5	0.5	4.42	0.0142	0.092	0.015	0.128	0.9138	0.040

1.1 Analysis of extracted sol (^{1}H-NMR)

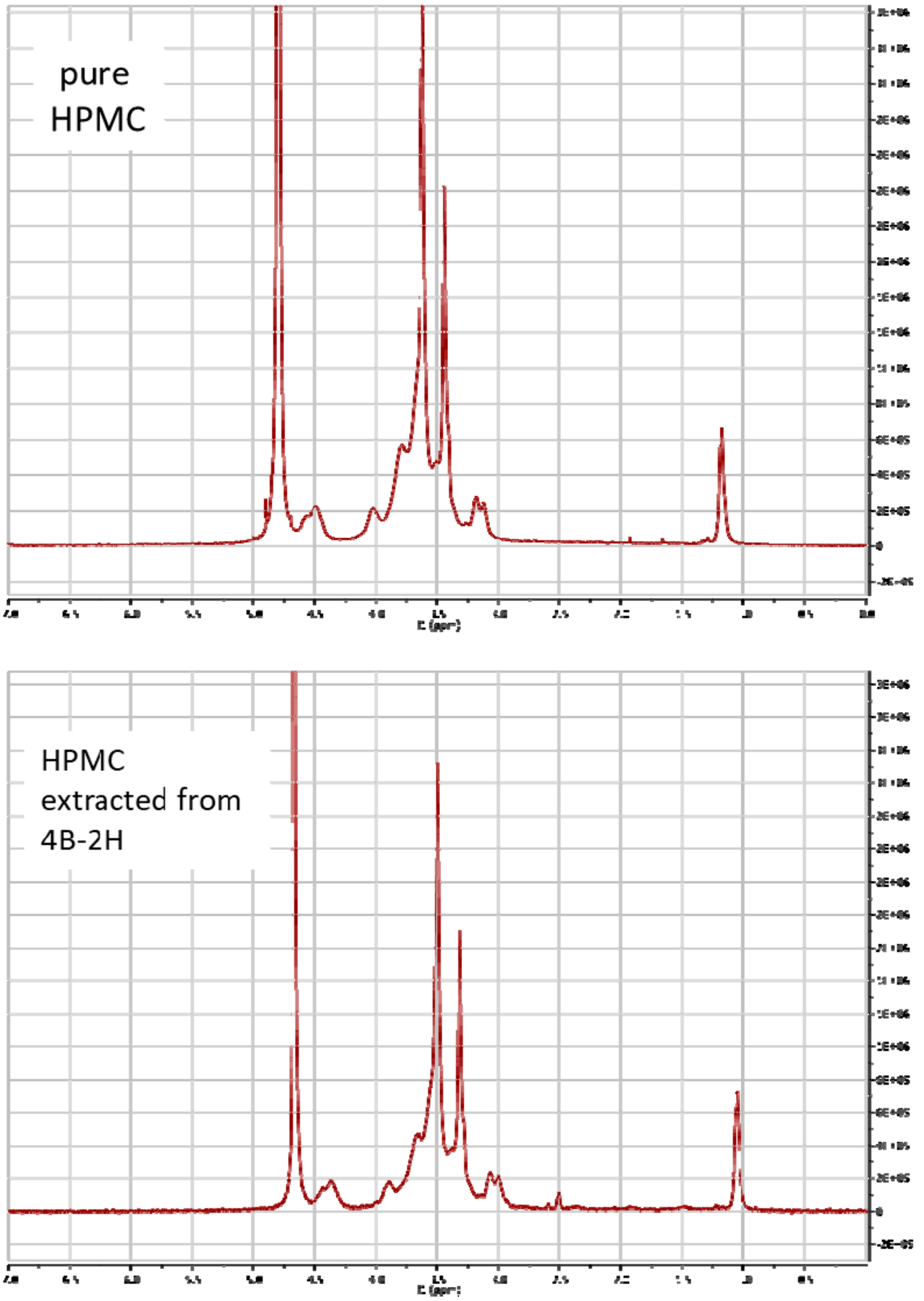

Figure S1 *1H-NMR spectrum of pure HPMC used in this work, and of the extract from 4B-2H: both spectra are identical.*

Table S2 *Sol extraction from the prepared gels.*

sample	sol fraction * (%)	fixed fraction of HPMC * (%)	residual HPMC content in dry gel (%)
1B	0	0	0
1B-1H	99	1	0.12
1B-2H	99	1	0.24
1B-3H	98	2	0.73
1B-5H	97	3	1.83
2B	0	0	0
2B-1H	99	1	0.12
2B-2H	99	1	0.24
2B-3H	97	3	1.09
2B-5H	97	3	1.84
4B	0	0	0
4B-1H	99	1	0.12
4B-2H	98	2	0.48
4B-3H	97	3	1.09
4B-5H	95	5	3.04

*) accuracy: ± 2%

2 Morphology of the prepared hydrogels

2.1 HPMC migration during drying, FTIR analysis of the extracted phase

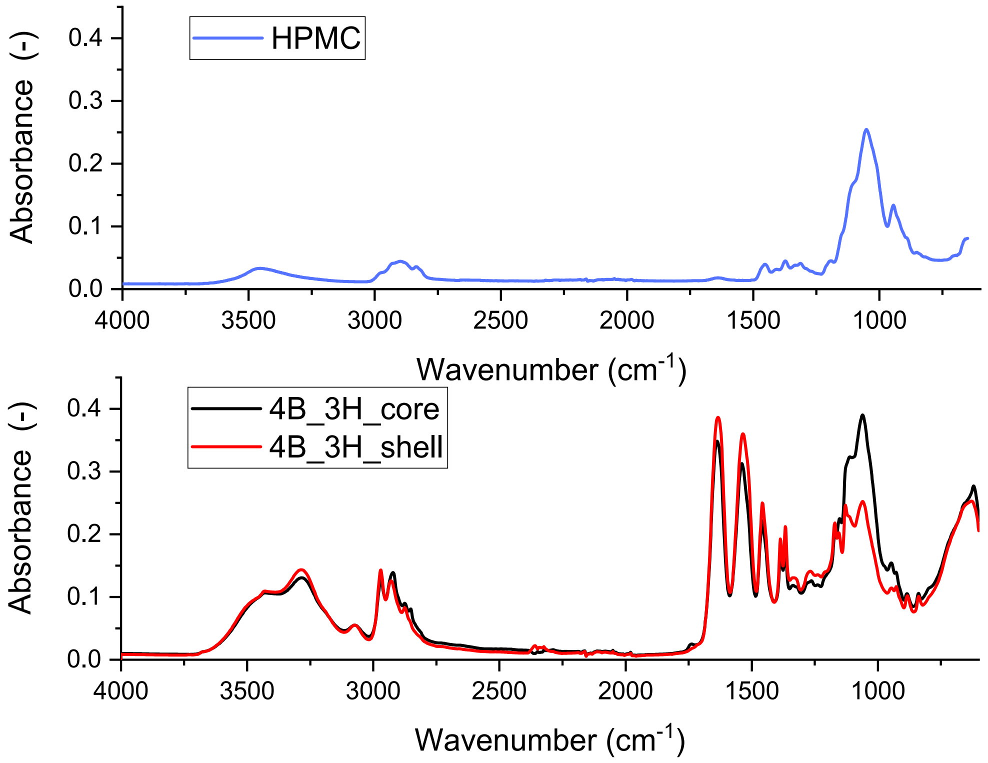

Figure S2 *FT-IR spectra of the dried sample 4B-3H-ap (state after preparation), namely of the core and of the outer layer; FTIR of pure HPMC also is shown for comparison.*

3 Moduli of the hydrogels

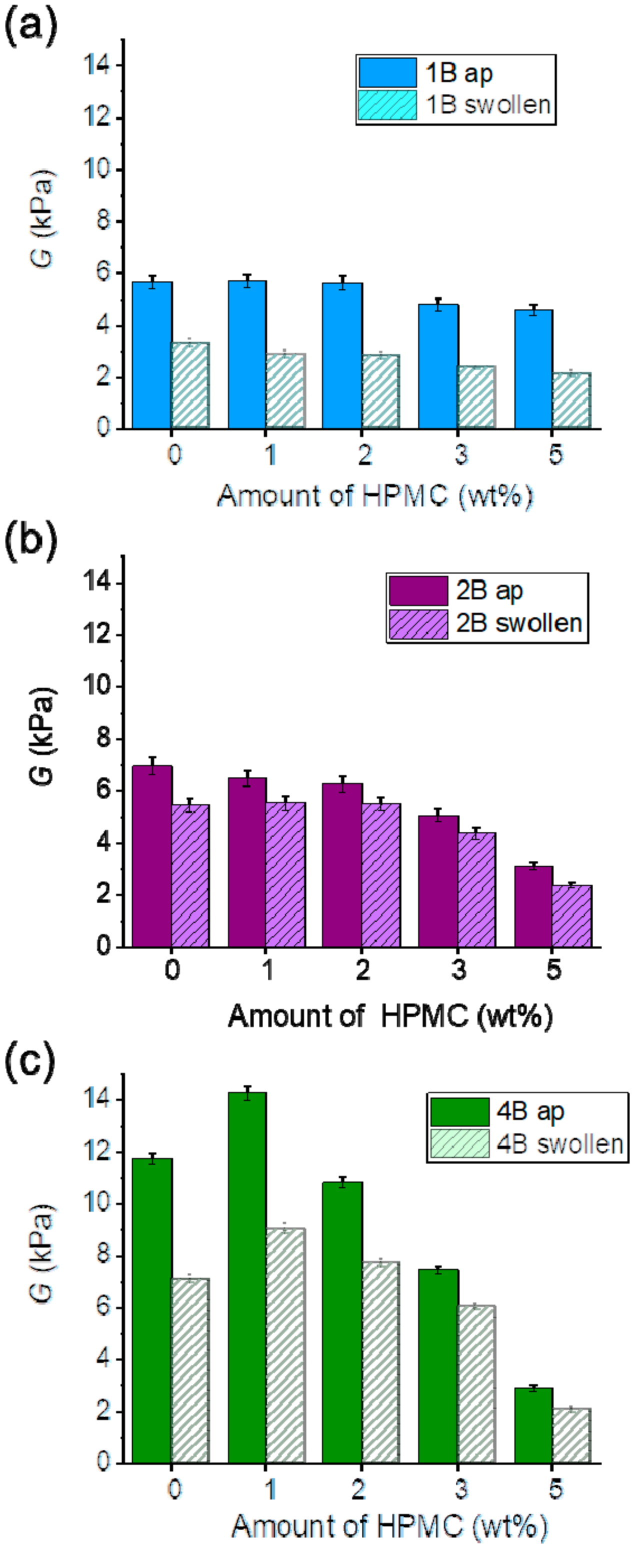

Figure S3 *Shear moduli of the PNIPAm/HPMC hydrogels series: (a) 1B series crosslinked by 1 mol% of BAA; (b) 2B series containing 2 mol% of BAA; (c) 4B series containing 4 mol% of BAA; in each series, full columns symbolize as-prepared gels, the shaded ones the equilibrium-swollen gels.*

3.1 Crosslinker amount

The effect of the crosslinker (BAA) content is distinct in both states of the products: In the range of the tested HPMC concentrations from 0 to 3%, the moduli increase if the BAA content increases. In case of the 'as-prepared' ("ap") samples (see **Figure S3** or main Manuscript file: **Figure 5a**), the trend is distinctly non-linear, with a small difference between 1 and 2 mol% of BAA and a great difference between 2 and 4 mol% of the crosslinker. In case of the swollen ("sw") state (**Figure 5b**), the trend is close to linear, which would be expected in the ideal case. The difference between the trends in "ap" and "sw" samples could be attributed to temporary PNIPAm chain entanglements, which in the less-swollen "ap" state could be more prominent than in the "sw" state, if the BAA content is low (long elastic chain segments). An exception from the above trends are the samples containing the maximum HPMC content, namely 5 wt.% (during synthesis and in the "ap" state). Here, the moduli either decrease with BAA content (in the "ap" state, especially strongly between 1 and 2 mol% BAA), or are indifferent to BAA content (in the "sw" state). This can be attributed to micro-phase separation, which increases if going from the sample 1B-5H (homogeneous) to 2B-5H and 4B-5H (both highly opaque), and which weakens the heterogeneous gels.

3.2 HPMC loading

The effects of the HPMC loading on the moduli of the hydrogels are very similar in the "ap" and in the "sw" state (see **Figure S3**). In the series with lower contents of crosslinker, the moduli (both "ap" and "sw") at first practically do not change with the increasing content of HPMC, while they somewhat drop at the highest HPMC contents. This final decrease is fairly small in the 1B series (crosslinked with 1 mol% of BAA) in which all the gels are homogeneous. The decrease might be attributed to a 'dilution effect' caused by the intercalated loosely bonded HPMC phase. In case of the 2B series, the effect of micro-phase separation ("ap" state) or of the empty spaces after extraction of the HPMC domains ("sw" state) additionally seems to cause a stronger modulus drop at the highest HPMC loadings. In case of the most strongly crosslinked 4B series, the reinforcing effect of hydrogen bonding between PNIPAm and HPMC eventually becomes distinct, but also the micro-phase-separation effects are most dramatic in this series. Hence, the modulus at first markedly rises if going from 4B to 4B-1H, after which it decreases again, eventually reaching ca. the same value like in the neat matrix (4B-ap or 4B-sw), as illustrated by modulus values of 4B-2H-ap and of 4B-3H-sw. Finally, at 5 wt.% of HPMC, the moduli of the PNIPAm/HPMC gels are markedly lower than in the neat 4B matrix, both in the "ap" and in the "sw" state.

The observed moderate mechanical reinforcing effect of HPMC can be attributed to hydrogen bonding PNIPAm–HPMC. In the "ap" state, the effect of HPMC content on the moduli in the 1B and 2B series is of similar strength, like the filler effect in the case of the previously studied non-porous PNIPAm/starch hydrogels [25]. In the latter systems, the filler phase was practically unextractable. Filler loadings also were similar in the mentioned PNIPAm/starch products and in the presently studied PNIPAm/HPMC-"ap" gels. In both types of materials, the intercalated phase causes (for sterical reasons) a decrease in covalent crosslinking density which, on the other hand, is practically compensated by the newly formed hydrogen bonding (as deduced in [25]). The considerable strength of the H-bonding in the PNIPAm/starch gels was demonstrated, however, if a secondary filler was added, which formed very strong H-bonds both to starch and to PNIPAm [25]. A similar demonstration of H-bond-strength was done with PNIPAm/starch gels which were shrunken at elevated temperature and subsequently left to cool down in the shrunken state [26].

The prominence of hydrogen bonding in the presently studied gels also becomes well-visible, if the moduli are carefully compared in the "ap" vs. the "sw" state for the series 1B, 2B and 4B (see **Figure S3**). Especially in case of 2B, the small difference is well-visible between the moduli in "ap" and "sw" state,

albeit the swelling degrees in these states vastly differed, namely by factors ranging from 1.8 (2B-1H) to 3.6 (2B-5H) see **Table 1** in the main Manuscript file. The moduli of the 2B series in the "sw" state hence ought to be distinctly smaller, if H-bonding were absent.

3.3 Changes in the nominal number of crosslinks (ratio r_1 / r_2)

Changes in the nominal number of crosslinks per specimen, which provide information about the strength of H-bonding between the PNIPAm network and the intercalated HPMC phase, can be evaluated by comparing moduli in the "ap" and "sw" state in view of their respective swelling degrees (see data in **Table 1** and **Figure 5c** in the main Manuscript file).

3.3.1 Mathematical evaluation

In the idealized case, the relation between the shear modulus (G) and the concentration of elastically active chains (cEAC) (= "crosslinking density") can be expressed by the formula:

$$G = c\text{EAC} \times R \times T \qquad \text{(s1)}$$

where R is the universal gas constant, and T is the temperature.
Per definition of concentration,

$$c\text{EAC} = n\text{EAC} / V \qquad \text{(s2)}$$

where nEAC is the molar amount of elastically active chains in the specimen, while V is the volume of the specimen.
The swelling degree (Q) of the samples was defined as:

$$Q = m_{\text{swollen, actual}} / m_{\text{dry}} \quad = \quad V_{\text{actual}} \times \rho_{\text{actual}} / m_{\text{dry}} \qquad \text{(s3)}$$

where m are the respective masses, and ρ_{actual} is the actual density of the specimen. In case of the hydrogels in both "ap" and "sw" state, the swelling degrees are high (always higher than 7), so that ρ_{actual} approximately is constant and equal to the density of pure water. The value of m_{dry} also is constant for a given sample in different swelling states (e.g. with swelling degrees Q_{ap} and Q_{sw}).
Hence it holds:

$$Q_{\text{eq}} / Q_{\text{ap}} = V_{\text{eq}} / V_{\text{ap}} \qquad \text{(s4)}$$

From the equations (s1) and (s2) it follows, that G is proportional to $1/V$ for a given specimen which occurs in different swelling states, and in which the molar amount of elastically active chains (nEAC) does not change.

From equations (s1), (s2) and (s4) it follows:

$$\boldsymbol{G_{\text{eq}} / G_{\text{ap}}} = c\text{EAC}_{\text{eq}} / c\text{EAC}_{\text{ap}} = V_{\text{ap}} / V_{\text{eq}} \quad \boldsymbol{= Q_{\text{ap}} / Q_{\text{eq}}} \qquad \text{(s5)}$$

as far as the number of crosslinks does not change.

In **Table 1** (main Manuscript), the ratios r_1 (= $Q_{\text{eq}} / Q_{\text{ap}}$) and r_2 (= $G_{\text{ap}} / G_{\text{eq}}$) are shown. In the case that the number (or molar amount) of elastically active chains in the specimen, nEAC, does not change during the swelling from the "ap" state up to the "sw" state, and that the idealized equation (1) holds, r_1 should be equal to r_2.
In case that $r_1 > r_2$, the modulus decreases less than expected according to equation (5). This would mean that additional crosslinks (including reversible physical ones) are formed in the equilibrium swollen state "sw".
The opposite case, $r_1 < r_2$, would mean a decrease of the absolute number of crosslinks in the sample (decrease of nEAC). The ratio r_1 / r_2 thus gives the relative increase of the number of crosslinks (or of the amount of elastically active chains).

The experimentally determined ratios r_1 / r_2 are shown in **Table 1** (main Manuscript), while their dependence on the amount of added HPMC and on the crosslinker content is analyzed in **Figure 5c** (main Manuscript).

3.3.2 Trends in the nominal number of crosslinks per specimen

It can be seen that the ratio r_1 / r_2 is greater than one in nearly all the tested samples, and that it distinctly increases with the content of HPMC present during the synthesis.

In case of neat PNIPAm crosslinked by 4 mol% BAA (sample "4B"), the ratio r_1 / r_2 is smaller than one. A similar but less marked situation is found for "4B-1H" (r_1 / r_2 = 0.9).

In case of the neat matrices (1B, 2B and 4B) the ratio r_1 / r_2 decreases with the increasing amount of crosslinker, from 1.3 down to 0.8 (especially strongly between 2B and 4B). The trend concerning the crosslinker can be tentatively explained by the effect of permanent entanglements which are more likely to form in case of long elastic chains between the crosslinks (e.g. in "1B"), combined with the effect of temporary (dissociation-able) entanglements which are more prominent in higher-crosslinked gels (e.g. in "4B"). Both the mutually opposed effects manifest themselves upon swelling.

Effect of BAA content: The ratio r_1 / r_2 distinctly increases (at any given HPMC content) if going from gels crosslinked with 1 mol% of BAA (1B series) to the 2B-series (except the neat matrices, where there is little change). This trend of r_1 / r_2 is a consequence of hydrogen bonding PNIPAm–HPMC, which in the higher-swollen state always leads to a higher chemical amount of elastically active crosslinks in a given sample (additional H-bonds). In case of the 2B series, additionally the higher density of the covalent crosslinks (more BAA), in combination with relatively moderate damage to the network structure caused by micro-phase-separation, leads to the highest relative increase in the amount of the elastically active chains (ratio r_1 / r_2) among the studied series. A record value of ratio r_1 / r_2 = 2.7 is reached by the gel 2B-5H, while the neat matrix 2B has the moderately non-ideal ratio r_1 / r_2 = 1.2. In contrast to this, the 4B-series always displays the smallest ratio r_1 / r_2 at all HPMC loadings, which is attributed to marked loosening of a network which otherwise would be tight by intercalation and nano-phase-separation of HPMC. Even so, the ratio r_1 / r_2 in the 4B series markedly increases if going from 4B to 4B-5H, similarly strongly like in the 2B series.

4 Tensile properties

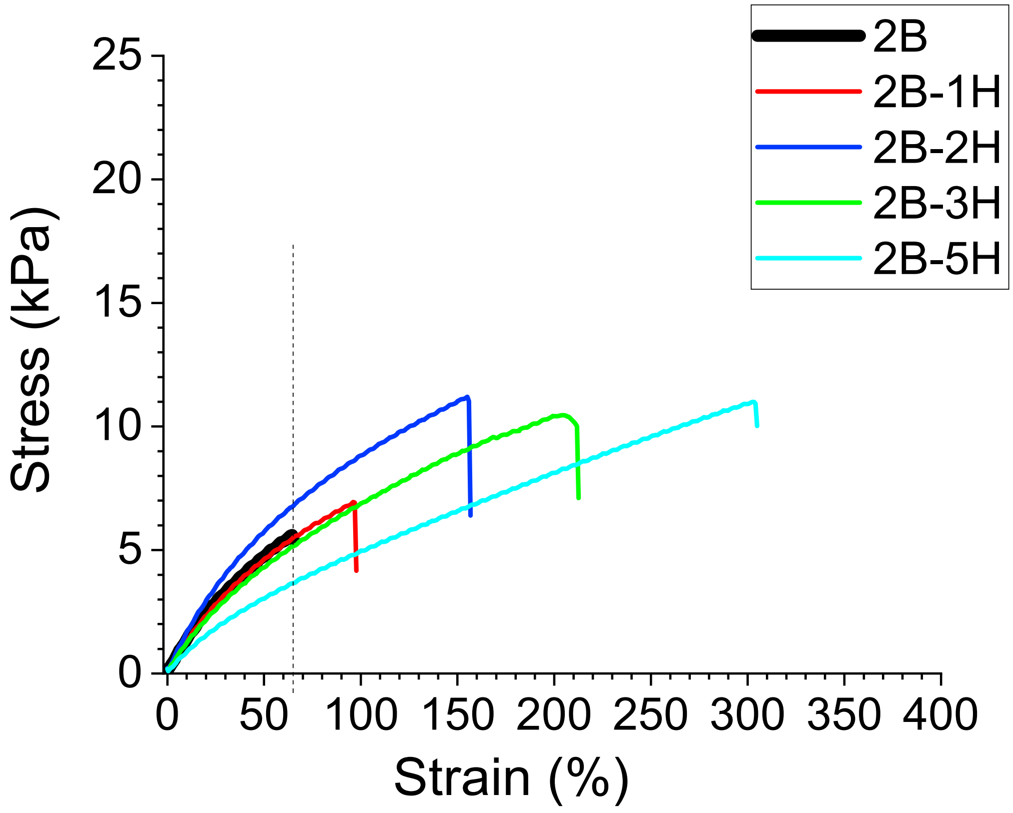

Figure S4 *Stress–strain curves of the 2B series (2 mol% of BAA crosslinker) of the PNIPAm/HPMC hydrogels in the after-preparation state.*

Table S3 *Tensile characteristics of the hydrogels.*

sample	Elongation at break (%)	Tensile strength (kPa)	Tensile toughness (kJ/m^3)
1B	100.2	5.54	3.40
1B-1H	137.3	7.37	6.1
1B-2H	167.5	7.80	8.1
1B-3H	250.8	9.16	14.3
1B-5H	376	9.81	24.2
2B	64.6	5.62	2.17
2B-1H	97.2	6.83	4.12
2B-2H	155.8	11.15	11.04
2B-3H	211.6	10.41	14.14
2B-5H	304	10.96	19.54
4B	34.8	6.43	1.4
4B-1H	130.7	16.46	14.7
4B-2H	165.8	19.16	19.3
4B-3H	250.4	17.25	29
4B-5H	203.3	9.90	10.1
4B-sw	25.8	6.31	1.00
4B-1H-sw	112.6	22.7	16.5
4B-3H-sw	158.3	16.6	17.1
4B-5H-sw	250.0	10.7	18.5

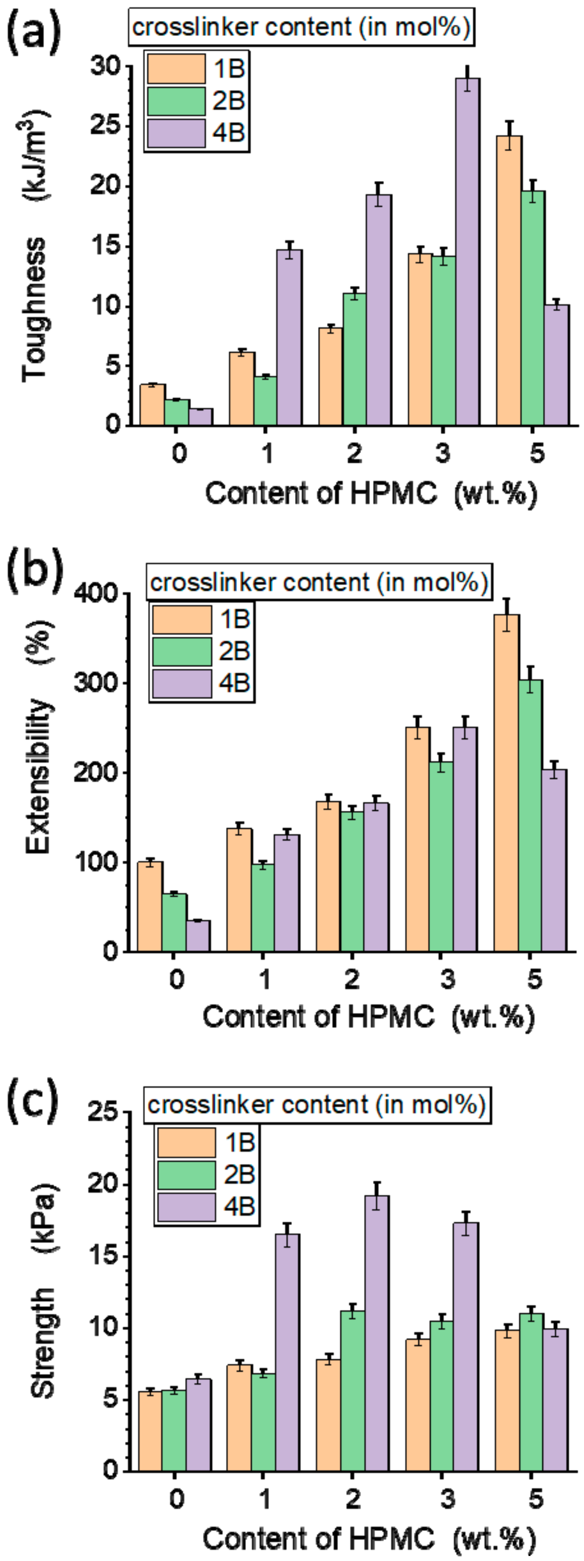

Figure S5 *Tensile characteristics obtained from the stress–strain curves of the studied PNIPAm/HPMC hydrogels in the after-preparation state: effect of composition on: (a) toughness, (b) extensibility, (c) tensile strength.*

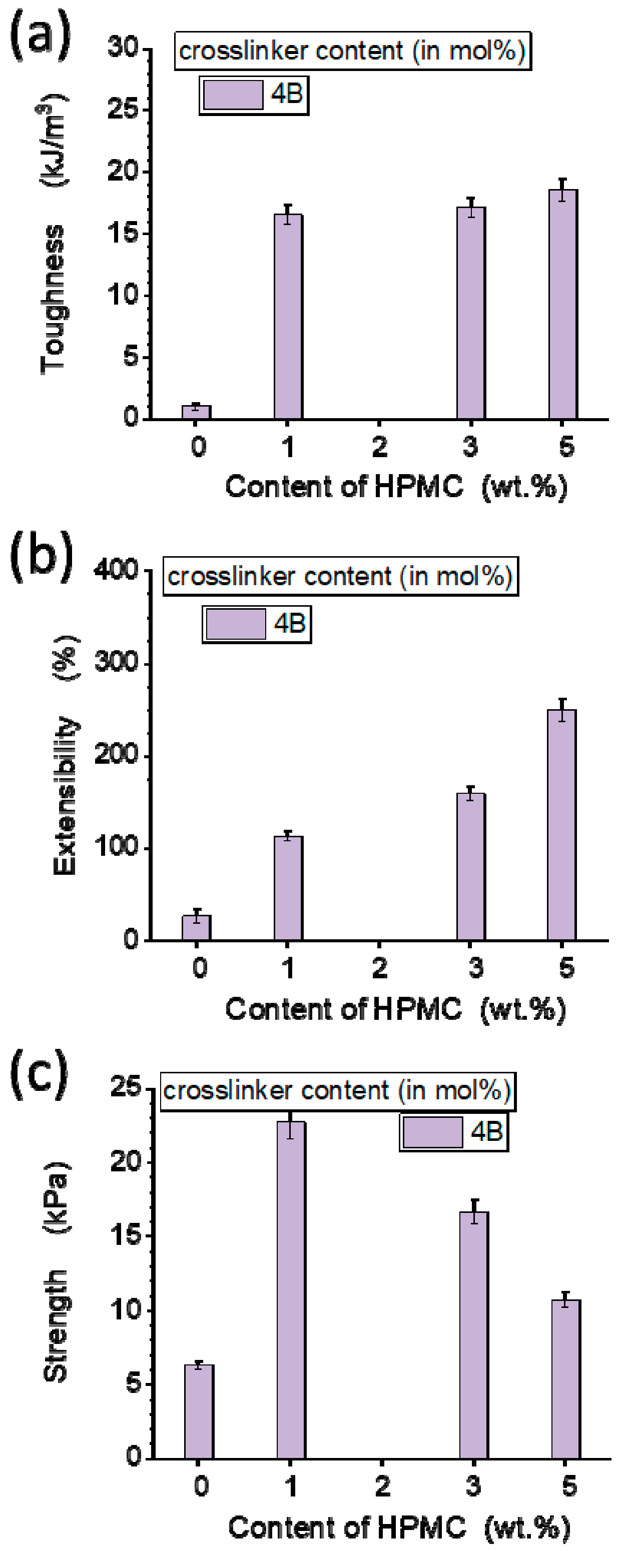

Figure S6 *Tensile characteristics obtained from the stress–strain curves of the equilibrium-swollen gels from the 4B series: effect of HPMC content on: (a) toughness, (b) extensibility, (c) tensile strength.*

4.1 Crushing tests

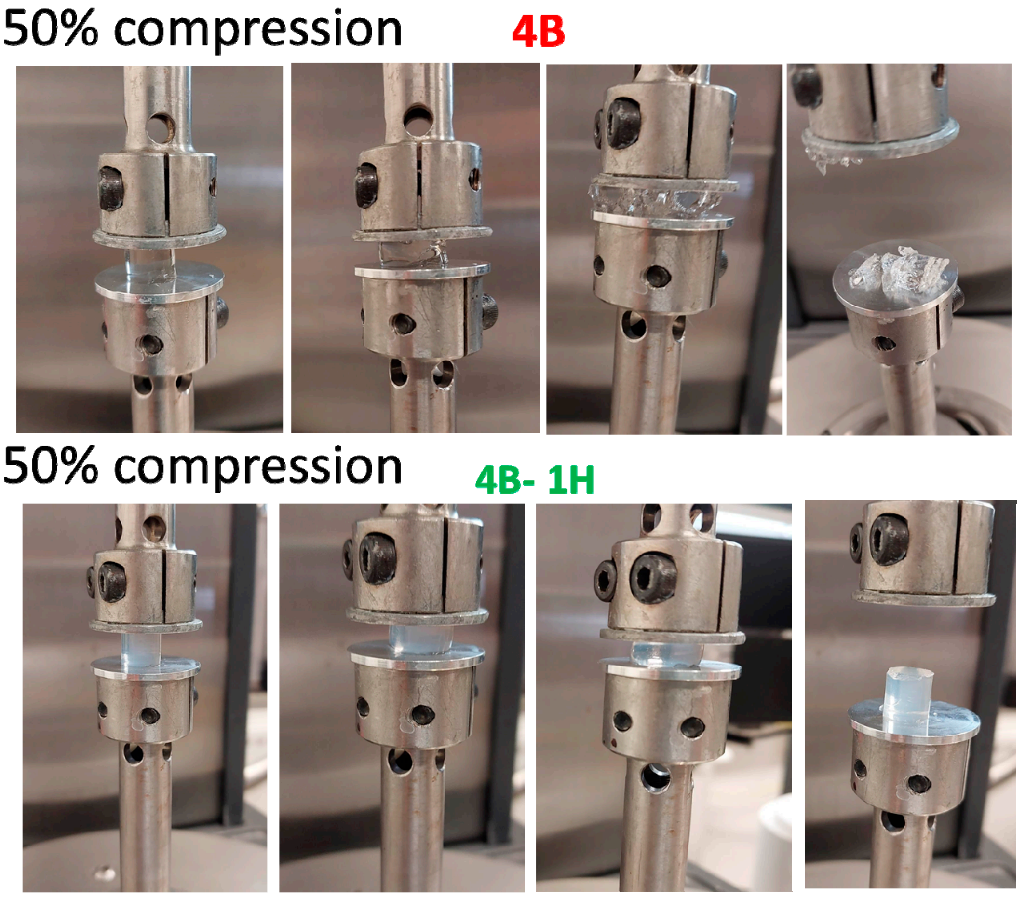

Figure S7 *Results of qualitative compressive ('crushing') tests performed until 50% of compression, with the samples 4B (neat PNIPAm) and 4B 1H.*

5 Temperature response

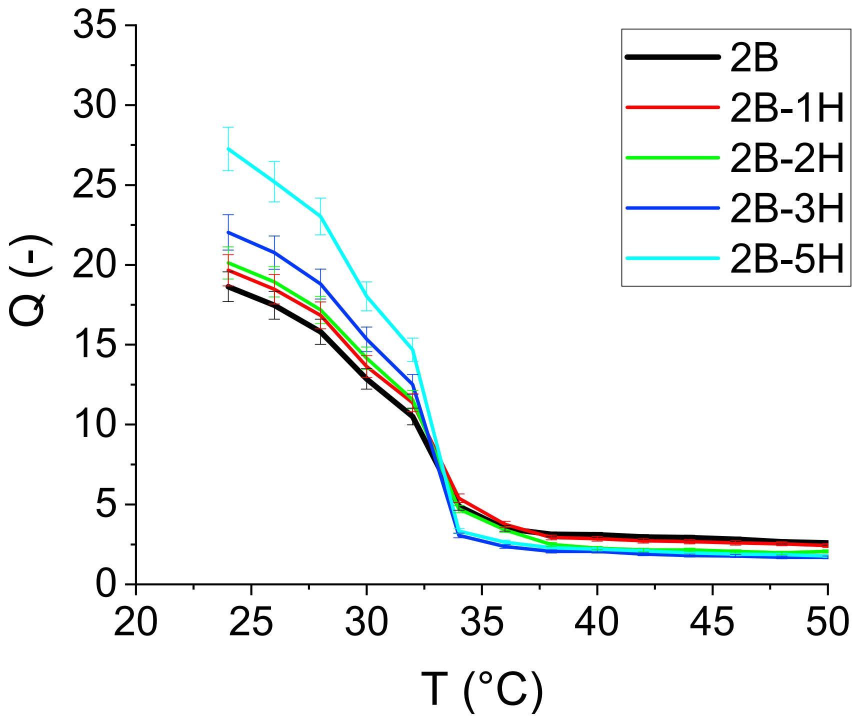

Figure S8 *Temperature dependence of the swelling degree (Q = f(T)) of the "2B" series of the PNIPAm/HPMC, which is crosslinked with 2 mol% of BAA: comparison of curves of gels with different HPMC contents.*

5.1 Kinetics of T-response

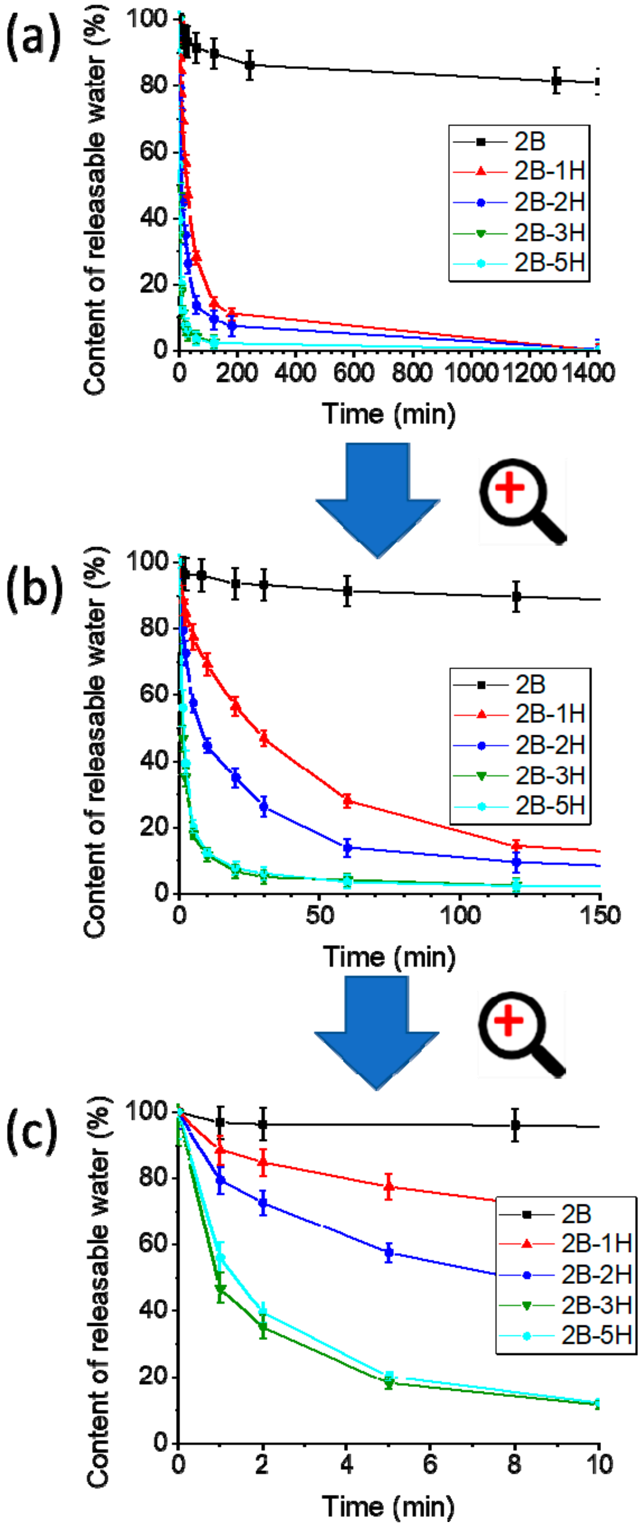

Figure S9 *Kinetics of shrinking: Relative contents of releasable swelling water as function of shrinking time after the temperature jump 25 → 50 °C: gels from the "2B" series crosslinked with 2 mol% of BAA and intercalated with 1 to 5% HPMC: (a,b,c) different time zooms.*

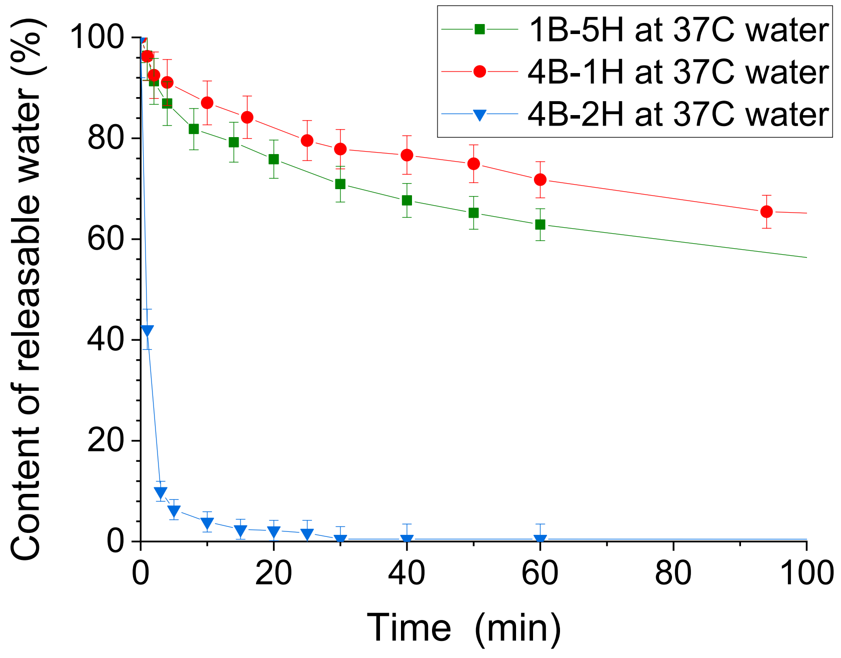

Figure S10 *Kinetics of shrinking: Relative contents of releasable swelling water as function of shrinking time after the temperature jump 25 → 37 °C: gels 1B-5H, 4B-1H, 4B-2H.*

6 Drug-release kinetics

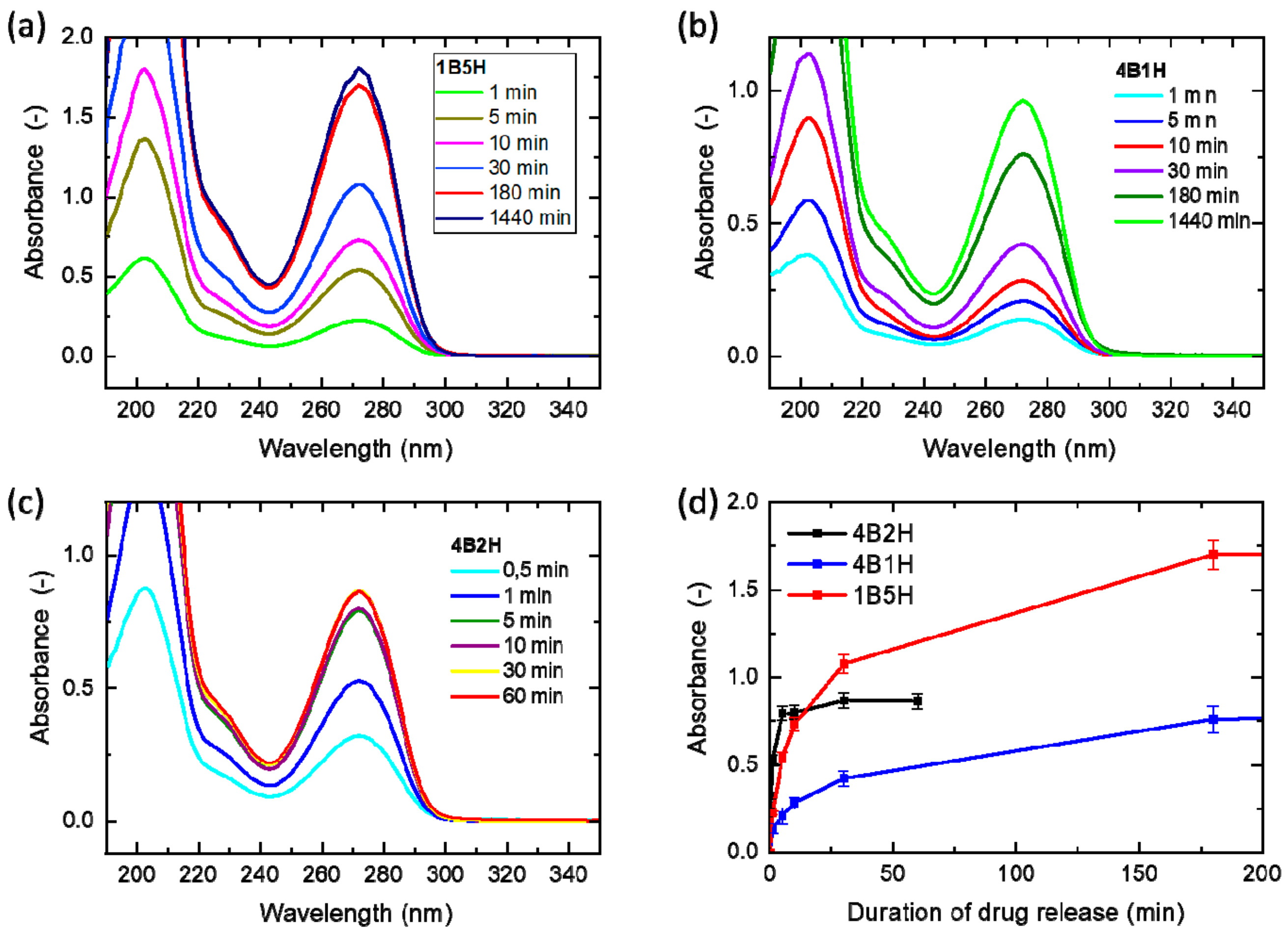

Figure S11 *Raw data for the drug release kinetics: UV/vis absorption spectra of 3-ml-specimens taken at different times from the 250 ml bath at 37 °C, into which the studied samples impregnated with Theophylline were releasing this drug: (a) 1B-5H as the Theophylline-impregnated sample, (b) 4B-1H as the impregnated sample, (c) 4B-2H as the impregnated samples, (d) time-dependent absorbance of Theophylline at the 272 nm for the drug-release-baths of the three tested gels.*

6.1 Evaluation of the drug release kinetics

Drug loading in all samples

Table S4 *Theophylline loading into selected hydrogels.*

sample	$m_{swollen}$ at 25 °C (g)	$m_{deswollen}$ at 50 °C for 24h (g)	$m_{sdrug\text{-}loaded}$ with Theophylline*, 48h at 25 °C (g)	Volume uptake*, drug solution (mL)	n(drug uptake)* (mol)
1B-5H	1.3335	0.1510	1.244	1.093	3.93E-05
4B-1H	0.6500	0.1412	0.6311	0.490	1.76E-05
4B-2H	0.6740	0.0781	0.6703	0.592	2.13E-05

*) the shrunken specimens were put into a Theophylline solution of 0.036 mol/L (volume: 15mL), whose density was 1.00 g/mL; the volume uptake was calculated from the mass uptake; the molar uptake of Theophylline was calculated according to $n = c \times V$.

Drug release

The extinction coefficient of Theophylline ε(272 nm) at the absorption maximum of 272 nm was measured to be 10 300 ±3% $(mol/L)^{-1}$ cm^{-1}.

In the columns of the evaluation tables, the following magnitudes are calculated:

At first, the actual concentration in the bath determined for every data point:

Concentrations of Theophylline solutions (c) were calculated from the measured absorbance (A) values (at 272 nm) according to Lambert-Beer: $c = A / (\varepsilon \times d)$, where d was the internal thickness of the cuvette.
The diminishing volume of the bath was calculated, taking into account every removed solution specimen (in the given case, every specimen were precisely 3 mL).
From $c_{in\ bath}$ and $V_{bath,\ actual}$ the molar amount of Theophylline present in the bath ($n_{drug\ in\ bath}$) is calculated ($n = c \times V$). Additionally, from the removed specimen volume (3 mL) and from the actual concentration of the removed bath solution ($c_{in\ bath}$), the small amount of Theophylline which was removed from the bath ($n_{drug,\ removed\ in\ step}$) also is calculated.

Next, the summarized molar amount of Theophylline which was removed from the bath until before the actual sampling step (table row) is calculated ($n_{drug,\ removed\ from\ bath,\ in\ total}$).
Finally, the total molar amount of released Theophylline at a given time point ($n_{drug\_RELEASED\ by\ gel,\ total}$) is summarized by adding $n_{drug\ in\ bath}$ and $n_{drug,\ removed\ from\ bath,\ summarized}$. The percentage of drug release is calculated the released amount ($n_{drug\_RELEASED\ by\ gel,\ total}$) and the initially loaded amount of Theophylline.

sample: **1B-5H**
n(drug, loaded) = 3.935E-05 mol
V(release bath): 250 mL
V(step = removed specimen): 3 mL

Table S5 *Release of Theophylline from 1B-5H.*

t (min)	A (-)	$c_{in\ bath}$ (mol/L)	Volume of bath, actual (L)	$n_{drug\ in\ bath}$ (mol)	Volume removed in step (L)	$n_{drug,}$ removed in step (mol)	$n_{drug,}$ removed from bath, summarized (mol)	$n_{drug\_RELEASED}$ by gel, total (mol)	Drug release (%)
0	0	0	0.250	0	0	0	0	0	0
1	0.203	1.97E-05	0.250	4.92E-06	0.003	5.90E-08	5.90E-08	4.92E-06	12.5
5	0.484	4.69E-05	0.247	1.16E-05	0.003	1.41E-07	2.00E-07	1.16E-05	29.6
10	0.653	6.32E-05	0.244	1.54E-05	0.003	1.90E-07	3.89E-07	1.56E-05	39.7
30	0.96	9.31E-05	0.241	2.24E-05	0.003	2.79E-07	6.68E-07	2.28E-05	58.0
180	1.52	1.47E-04	0.238	3.50E-05	0.003	4.41E-07	1.11E-06	3.57E-05	90.6
1440	1.61	1.56E-04	0.235	3.67E-05	0.003	4.69E-07	1.58E-06	3.78E-05	96.2

sample: **4B-1H**
n(drug, loaded) = 1.764E-05 mol
V(bath): 250 mL
V(step): 3 mL

Table S6 *Release of Theophylline from 4B-1H.*

t (min)	A (-)	$c_{in\ bath}$ (mol/L)	Volume of bath, actual (L)	$n_{drug\ in\ bath}$ (mol)	Volume removed in step (L)	$n_{drug,}$ removed in step (mol)	$n_{drug,}$ removed from bath, summarized (mol)	$n_{drug\_RELEASED}$ by gel, total (mol)	Drug release (%)
0	0	0	0.25	0	0	0	0	0	0
1	0.104	1.01E-05	0.25	2.52E-06	0.003	3.02E-08	3.02E-08	2.52E-06	14.3
5	0.156	1.51E-05	0.247	3.73E-06	0.003	4.53E-08	7.55E-08	3.76E-06	21.3
10	0.211	2.04E-05	0.244	4.98E-06	0.003	6.12E-08	1.37E-07	5.06E-06	28.7
30	0.315	3.05E-05	0.241	7.36E-06	0.003	9.16E-08	2.28E-07	7.50E-06	42.5
180	0.567	5.49E-05	0.238	1.31E-05	0.003	1.65E-07	3.93E-07	1.33E-05	75.4
1440	0.72	6.96E-05	0.235	1.64E-05	0.003	2.09E-07	6.02E-07	1.67E-05	95.0

sample: **4B-2H**
n(drug, loaded) = 2.131E-05 mol
V(bath): 250 mL
V(step): 3 mL

Table S7 *Release of Theophylline from 4B-2H.*

t (min)	A (-)	$c_{\text{in bath}}$ (mol/L)	Volume of bath, actual (L)	$n_{\text{drug in bath}}$ (mol)	Volume removed in step (L)	$n_{\text{drug, removed in step}}$ (mol)	$n_{\text{drug, removed from bath, summarized}}$ (mol)	$n_{\text{drug\_RELEASED by gel, total}}$ (mol)	Drug release (%)
0	0	0	0.25	0	0	0	0	0	0
0.5	0.321	3.11E-05	0.250	7.78E-06	0.003	9.33E-08	9.33E-08	7.78E-06	36.5
1	0.529	5.12E-05	0.247	1.27E-05	0.003	1.54E-07	2.47E-07	1.28E-05	59.8
5	0.794	7.69E-05	0.244	1.88E-05	0.003	2.31E-07	4.78E-07	1.90E-05	89.2
10	0.80	7.74E-05	0.241	1.86E-05	0.003	2.32E-07	7.10E-07	1.91E-05	89.7
30	0.87	8.40E-05	0.238	2.00E-05	0.003	2.52E-07	9.62E-07	2.07E-05	97.2
60	0.86	8.37E-05	0.235	1.97E-05	0.003	2.51E-07	1.21E-06	2.06E-05	96.8

Literature References from the Main Manuscript which are cited

[25] Depa, K., Strachota, A., Šlouf, M., & Brus, J. (2017). Poly(N-isopropylacrylamide)-SiO2 nanocomposites interpenetrated by starch: Stimuli-responsive hydrogels with attractive tensile properties. *European Polymer Journal*, *88*, 349–372. https://doi.org/10.1016/j.eurpolymj.2017.01.038

[26] Strachota, B., Strachota, A., Šlouf, M., Brus, J., & Cimrová, V. (2019). Monolithic intercalated PNIPAm/starch hydrogels with very fast and extensive one-way volume and swelling responses to temperature and pH: prospective actuators and drug release systems. *Soft Matter*, *15*(4), 752–769. https://doi.org/10.1039/c8sm02153h

List of Figures and Schemes from the main manuscript

Figure 1. Swelling degrees of the hydrogels after preparation and in the equilibrium-swollen state; *) ‘as prepared’ state: same values for all three series.

Figure 2. Appearance of the prepared PNIPAm/HPMC hydrogels, in equilibrium-swollen state.

Figure 3. Large-scale morphology (as observed by light microscopy) of samples in the ‘as prepared’ state (a, b), and in the swollen state (c-h); comparison of the effect of different concentration of BAA and of HPMC.

Figure 4. Morphology of hydrogels which were dried: (a–c) from the ‘as prepared’ state: sample 2B 5H, or (d–h) from the equilibrium swollen state: sample 2B 5H swollen (d–f) and sample 4B-5H-swollen (g,h); optical photographs of the dried gels are shown in (a) and (d).

Figure 5. Shear moduli of the PNIPAm/HPMC hydrogels: (a) after preparation; (b) in equilibrium swollen state; (c) the ratios of change of the amount of crosslinks during swelling.

Figure 6. Stress–strain curves of the hydrogels: (a,b) after preparation and (c) in the swollen state.

Figure 7. Result of qualitative compressive (‘crushing’) test performed until 80% of compression, with the sample 4B 5H.

Figure 8. Temperature dependence of the swelling degree (Q = f(T)) of the PNIPAm/HPMC gels for (a) 1 mol% BAA and for (b) 4 mol% BAA; HPMC content is indicated in the legend.

Figure 9. Appearance of selected hydrogels during the deswelling process.

Figure 10. Kinetics of shrinking: Relative contents of releasable swelling water as function of shrinking time after the temperature jump 25-->50 °C: (a,b) gels from “1B” series crosslinked with 1 mol% of BAA and intercalated with 1 to 5% HPMC at different time zoom; (c,d) “4B” series containing 4 mol% BAA + 1–5% HPMC; (e) shrinking factors for all the tested hydrogels (Q at 25°C / Q at 50 °C).

Figure 11. Kinetics of shrinking of an exemplary sample, 4B 2H ap, in the as-prepared state: Relative contents of releasable swelling water as function of shrinking time after the temperature jump 25 --> 50 °C.

Figure 12. Kinetics of the release Theophylline: (a) Structure of the drug Theophylline. (b) Time-dependent UV/vis spectra of the water bath (at 37 °C) into which the drug Theophylline was released by the 1B-5H gel; the peak (272 nm) used for photometric determination of concentration is highlighted. (c) Percentage of drug release at 37 °C as function of time, for samples indicated in the legend. (d) Completion in % of a simple (drug-free) deswelling process in response to the T-jump from 25 to 37 °C, expressed in %, shown for the same gels which were tested as drug release materials.

Scheme 1 Synthesis of the gels: (a) chemistry; (b) intercalation of HPMC into PNIPAm network.

Scheme 2 Swelling of the prepared hydrogels and extraction of HPMC macromolecules which are not per-manently fixed.

Scheme 3 The crosslinked structure of the gels, and the effect of embedded HPMC.

Scheme 4 Interactions between PNIPAm and HPMC: (a) hydrogen bonding possibilities; (b) additional crosslinking generated by H-bridges between PNIPAm and HPMC.

Scheme 5 The mechanism of fast shrinking: (a) supporting role of micro-phase separation; (b) molecular mechanism on PNIPAm/polysaccharide interface.

Table 1. Swelling degrees, moduli and concentrations of elastically active chains (cEAC) in its initial state (“ap” = ‘as prepared’) and after swelling to equilibrium.
